# Comparative corrosion behavior of three titanium alloy base materials and their welds in a simulated chimney environment

**DOI:** 10.1016/j.heliyon.2024.e36022

**Published:** 2024-08-10

**Authors:** Caigan Che, Xuhui Zhao, Yu Zuo, Yuming Tang

**Affiliations:** a BUCT, 100029, Beijing, China; b INSA-Rennes, 35700, Rennes, France

**Keywords:** Titanium alloys, Argon arc welding, Chimney flue gas, Sulfuric acid dew point corrosion, Electrochemical testing, Microstructure analysis

## Abstract

This study investigates the corrosion behavior of titanium alloys (TA2, TC4, TB6) in a 3 % sulfuric acid flue gas environment using electrochemical tests and microscopic analyses (SEM/EDS, XRD, metallographic microscopy). Results show that TA2 base metal has lower corrosion resistance compared to its weld metal, while TC4 and TB6 exhibit opposite trends. Specifically, TC4 and TB6 base metals have lower corrosion current densities (0.9 and 0.5 μA/cm^2^) and higher corrosion potentials then their weld metals (1.93 and 2 μA/cm^2^). In contrast, TA2 base metal showed higher corrosion current density (2 μA/cm^2^) than its weld metal (0.35 μA/cm^2^) and HAZ metal (0.16 μA/cm^2^).

Microscopic analyses reveal β phase transitions in TC4 and TB6 weld areas, leading to larger grain sizes and reduced corrosion resistance. Conversely, TA2 retains finer grains post-welding, enhancing its corrosion resistance. These insights clarify weld corrosion effects and provide valuable guidance for industrial applications of titanium alloys, particularly in designing and maintaining titanium alloy chimneys.

## 1 Introduction

Titanium, abundant in the Earth's crust, has gained significant attention across various industries due to its exceptional properties, including corrosion resistance, high specific strength, and suitability for low-temperature environments [1]. In particular, titanium and titanium alloys exhibit exceptional corrosion resistance in various environments, which is attributed to their ability to naturally form a thin and dense surface oxide layer [2]. However, in deaerated conditions or low pH reducing environments, the instability of the passive film compromises the metal integrity [3]. With recent decreases in titanium alloy prices, industries are increasingly adopting titanium alloys for applications traditionally dominated by materials such as iron and stainless steel. Therefore, despite its superior characteristics, titanium alloy chimneys still face corrosion challenges, particularly from acidic dew in flue gas environments.

Chimneys play a pivotal role in power generation, stabilizing production systems by safely discharging flue gas into the atmosphere at an appropriate height. The combustion of fossil fuels and bituminous coals generates sulfides, converting into corrosive compounds like SO_2_ and SO_3_ [4]. Therefore, understanding the corrosion behavior of titanium alloy chimney weldments is crucial. Furthermore, the workload of on-site processing, production, and welding of titanium plates is substantial, and ensuring weld quality is challenging. Therefore, performance defects such as crack defects often occur in the welding process, making welds generally more susceptible to corrosion.

However, previous research on the corrosion behavior of welded titanium alloy weldments has not reached a unified conclusion [[5], [6], [7], [8], [9], [10], [11]]. Some suggest better corrosion resistance in weld areas, while others report inferior corrosion resistance compared to base metals. For example, Luo et al. used electrochemical testing method to study the electrochemical corrosion behavior of TA2 base metal and welded joints in the chloride-containing solutions (3.5 % NaCl, 5.0 % NaCl, 7.5 % NaCl). The results show that compared with the base metal, the polarization resistance is larger and the corrosion current density is smaller, indicating that the corrosion resistance of the welded joint is better [5]. A similar point of view can also be found in Zhou [6] 's study of the corrosion behavior of TA2 titanium alloy in a 3.5 wt% NaCl solution showed that the corrosion resistance of the metal in the weld area (including the heat-affected zone near the weld) is better than that of the base metal.

Conversely, other studies challenge this perspective. Sun [7] studied the corrosion behavior of TA2 pure titanium plate after surfacing to simulate condensation. He found that the corrosion resistance of the weld is basically the same as that of the base metal, and the welding sequence has no effect on the corrosion resistance of the front and back of the weld. However, Zhang [8] et al. studied the corrosion behavior of TA2 titanium alloy welded joints in seawater at different temperatures. Conventional macroscopic electrochemical analysis results show that the heat-affected zone always has the best corrosion resistance, followed by the base metal and then the weld. The weld seam is more severely corroded than the base metal.

The discrepancy extends to studies by Wang et al. [9] who noted an exacerbation in the corrosion behavior of TC4 alloys subjected to galvanic corrosion post-immersion in artificial seawater. These findings are corroborated by Fossati [10], who posited that prolonged high temperatures and slow cooling rates during welding can result in an overheated structure with coarse grains, thereby diminishing the weld area's corrosion resistance. Heidarbeigy [11]attributed the reduced corrosion resistance of welds compared to base metals to solute segregation and chemical heterogeneity within the columnar grain structure of the weld, which lowers the corrosion potential.

These structural changes are profound during welding, with titanium alloy welded joints experiencing complete recrystallization within the fusion zone. This process brings about considerable alterations in microstructure, phase content, composition, and distribution, often resulting in joints that exhibit notably less corrosion resistance than their rolled or cast fine-grain base metal counterparts [12,13].

These discrepancies underscore the need for further investigation to clarify the corrosion behavior of titanium alloy weldments, especially in flue gas environments. This paper aims to address the corrosion challenges faced by titanium alloy chimneys.

Specific objectives are as follows:

✓ To investigate the corrosion behavior of three typical titanium alloys (TA2, TC4, and TB6) used in industrial chimneys.
✓ To analyze the differences in corrosion behavior between titanium chimney argon arc welding (ARC) welds and base metals.
✓ To utilize electrochemical measurement techniques, such as potentiodynamic polarization curves and electrochemical impedance spectroscopy (EIS), to assess corrosion behavior.
✓ To conduct microstructural analysis using metallographic microscopy, SEM, XRD, and EDS to characterize changes in base metal and weld structure, phase composition, and crystal direction-dependent corrosion.

By systematically analyzing the corrosion behavior of three representative titanium alloy weldments, this study aims to provide important theoretical insights and practical applications for extending the service life of titanium alloy weldments and enhancing their safety and performance in industrial settings.

## 2 Experimental

### 2.1 Sample and solution preparation

The three titanium alloy materials TA2, TB6, and TC4 used in the experiment were provided by Baoji Dexin Titanium Industry Co., Ltd. The dimensions were 60 mm × 30 mm × 5 mm. The chemical compositions of the three titanium materials are shown in Table 1.

**Table 1:** Chemical components of three kinds of titanium alloy (wt.%).

Grade	Name	Main ingredients/%			Impurities/%				
		Ti	Al	V	Fe	C	N	H	O
TA2	commercial pure titanium	margin	/	/	0.11	0.02	0.03	0.002	0.09
TB6	Ti–10 V–2Fe–3Al	margin	2.92	9.80	1.89	0.05	0.01	0.013	0.07
TC4	Ti–6Al–4V	margin	6.24	4.07	0.30	0.08	0.05	0.015	0.20

**Fig. 1:**
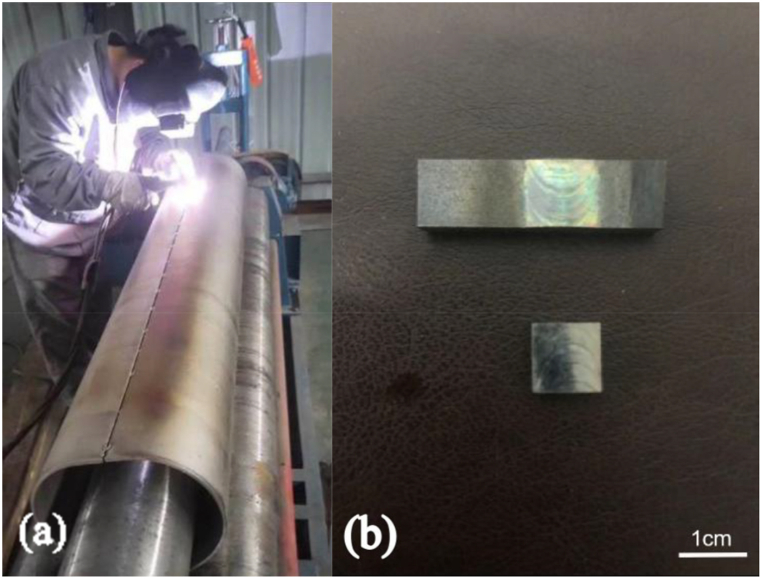
Actual drawing of manual argon arc welding (a) and physical drawing of the cut part of weld (b).

Three kinds of titanium alloy materials are welded by manual chimney argon arc welding (ARC), and DC forward connection is made by inverter arc welding machine (WS-7-315A). The main welding process parameters are shown in Table 2. First, the titanium plate was fixed by spot welding, with a 30-s delayed ventilation time at each solder joint. At the same time, the argon cylinder was opened, and a protective cover was used to protect the weld seam and heat-affected zone simultaneously. When starting the arc, gas was supplied in advance to produce high frequency arc; when extinguishing the arc, a current attenuation and gas protection device was used. After arc extinguishing, the welding wire is removed without protection, and the arc remains stable during this period. After welding, the surface was bright silver, indicating that the welding process is not affected by oxidation. The manual welding process and the resulting weld were examined by cutting a square sample of 1 cm^2^ from the weld part, as shown in Fig. 1.In order to simulate chimney flue gas environment within the dew point corrosion in titanium chimney, 3 wt% sulfuric acid solutions were selected as the acidic simulation liquid after different degrees of desulfurization. In the corrosion environment of titanium alloy chimneys, research by Rylands and Jenkinson [14] shows that the most important factor in the corrosion process is the deposition rate of sulfuric acid on the equipment surface, which depends on the difference between the dew point and the surface temperature. It was also found that the maximum deposition of sulfuric acid occurs when the surface temperature is 30–40 °C lower than the dew point temperature of the acid. Using the equation of Verchoff [15], as shown in (Equations (1)–(1)), the best corresponding relationship between the theoretical value of the dew point temperature and the measured value is obtained.

$$\frac{1}{T_{DP}}=\left(2.276\times 10^{-3}\right)-\left(2.943\times 10^{-5}\right)\ln\;p_{H_{2}O}-8.58\times 10^{-5})\ln\;p_{H_{2}SO_{4}}$$

(1-1) $$+\left(6.5\times 10^{-6}\right)\left(\ln\;p_{H_{2}O}\right)\left(\ln\;p_{H_{2}SO_{4}}\right)$$

**Table 2:** Manual argon arc welding process parameters.

Welding voltage/V	Welding current/A	Nozzle diameter/cm	Argon flow/(L min^1−)^		
			nozzle	Support cover	back
10	150	2	15	25	15

TDP - dew point temperature, K; PH_2_O - water pressure, mmHg; PH_2_SO_4_ - partial pressure of sulfuric acid, mmHg.

As the dew point temperature of steam is reached [15], when the surface temperature is lower than 50 °C, the condensation rate increases drastically, and the acidic flue gas condenses on the inner surface of the chimney, creating a harsh corrosive environment that causes significant corrosion to the titanium alloy material of the chimney.

S.Krakowiak [16] chose 1 % sulfuric acid solution to simulate the chimney flue gas environment. Removal of chloride and fluoride in the form of HCl or HF acid. Sun et al. [17]measured and obtained synchronous data on the waste gas flow rate and PM, SO_2_, NO_x_ and CO_2_ concentrations from the main emission sources of 22 steel plant sites on site. The highest emission concentration of PM, SO_2_, NO_X_ and CO_2_ were 38.12 mg/m^3^, 138.26 mg/m^3^, 217.28 mg/m^3^, 25.94 %, respectively. By comparison, it was found that the concentration of H_2_SO_4_ in the condensed solution in the flue gas after desulfurization is about 1–3 %, and considering that the temperature reaches the dew point corrosion, three types of titanium materials that are widely used in the industrial field and have excellent performance (Industrial pure titanium TA2, TC4, TB6) were tested in a 3 wt% H_2_SO_4_ flue gas simulation liquid.

### 2.2 Electrochemical measurements

All electrochemical measurements were carried out in a three-electrode electrochemical system using an electrochemical workstation (CS350H, China). The counter and reference electrodes were Pt plate and saturated calomel electrode (SCE), respectively.

**Fig. 2:**
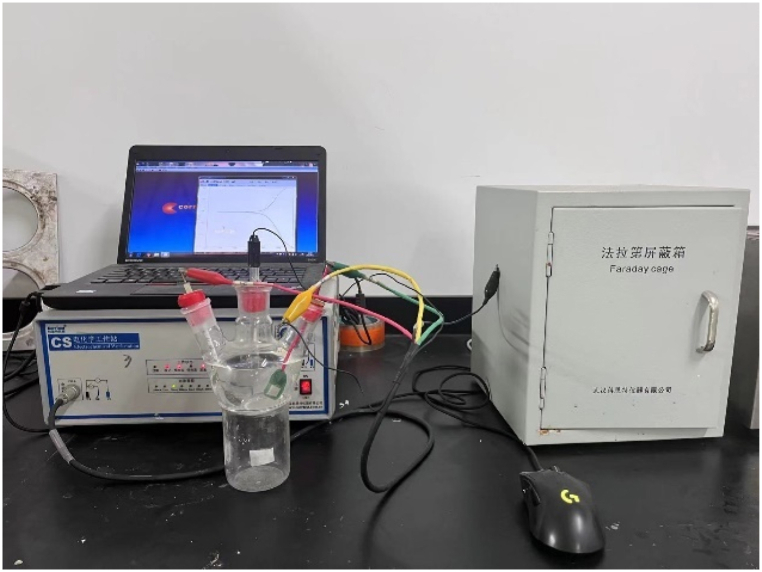
Corrosion test three-electrode system design illustration.

Electrochemical setup measurements experimental design is shown in Fig. 2. The three kinds of titanium alloys were embedded in epoxy resin leaving a working area of 10 mm × 10 mm to be as working electrodes, which were wet ground up to 2000 grit with a series of waterproof SiC abrasive papers and then ultrasonically cleaned with ethanol and deionized water, respectively, before electrochemical measurements. OCP measurements were stabilized for 4 h, and then potentiodynamic polarizations were conducted from −0.3 V to 2 V (versus OCP) at a scanning rate of 1 mV/s. EIS measurements were carried out at OCP using a sinusoidal potential perturbation of 10 mV in a frequency range from 100 kHz to 10 mHz. Before the EIS measurements, the OCPs were monitored for a period of time until the potential value became reasonably stable.

### 2.3 Characterisation

The phase constituents and element contents of base metal and weld metal from all types of titanium alloy samples were examined by X-ray diffraction (2500VB2, Bruker, Germany) using CuKα radiation with 2*θ* ranging from 30° to 80° at room temperature and tested by X-ray energy dispersive spectroscopy (EDS), respectively. An XTS-30 optical microscope and BCPCAS-4800 scanning electron microscope were used to further distinguish the microstructural differences between the base metal and weld metal of the three types of titanium alloy samples. For the microstructural studies of the alloy samples, the Kroll's solution composed of 10 ml HF, 15 ml HNO_3_, and 75 ml H_2_O was also prepared as the etchant solution. The solutions were prepared with analytical grade reagents and double-distilled water.

## 3 Results and discussion

### 3.1 Electrochemical studies

In this section, potential polarization tests and electrochemical impedance spectroscopy (EIS) were carried out on the three kinds of titanium alloy base metal metals and weld metals at ambient temperature using a 3 wt% H_2_SO_4_ flue gas simulation solution below the dew point temperature. Additionally, the equivalent circuit of electrochemical impedance spectroscopy is fitted and analyzed. The corrosion behavior of the three kinds of titanium alloy base materials and welds in different concentrations of H_2_SO_4_ flue gas simulation solution was compared and studied.

To simulate the chimney flue gas corrosion environment, a 3 wt% sulfuric acid solution was used at 25 °C. Potentiodynamic polarization tests were then performed on three titanium alloys: the base metals, the corresponding weld metals, and the TA2 heat-affected zone metals. Table 3 displays the passivation current (E _*pit*_), pitting potential (E _*pit*_) and corrosion potential (E _*corr*_)/current (I _*corr*_) data.

**Table 3:** Corrosion potential, passivation current and corrosion current of different titanium alloy base metal (BM), weld metal (WM) and TA2 heat-affected zone metal (HAZ) in 3 wt% H_2_SO_4_ flue gas corrosion simulation solution at 25 °C.

Sample	E _*corr*_ (V vs.SCE)	E _*pit*_ (V vs.SCE)	I _*pass*_ (μ A/cm^2^)	I _*corr*_ (μ A/cm^2^)
TA2 BM	−0.26	1.6	10.3	2
TA2 WM	−0.25	1.58	8.0	0.35
TA2 HAZ	−0.24	1.5	1.8	0.16
TC4 BM	−0.31	1.52	6.0	0.9
TC4 WM	−0.32	1.6	8.5	1.93
TB6 BM	−0.27	1.5	4.3	0.5
TB6 WM	−0.29	1.55	10.0	2

**Fig. 3:**
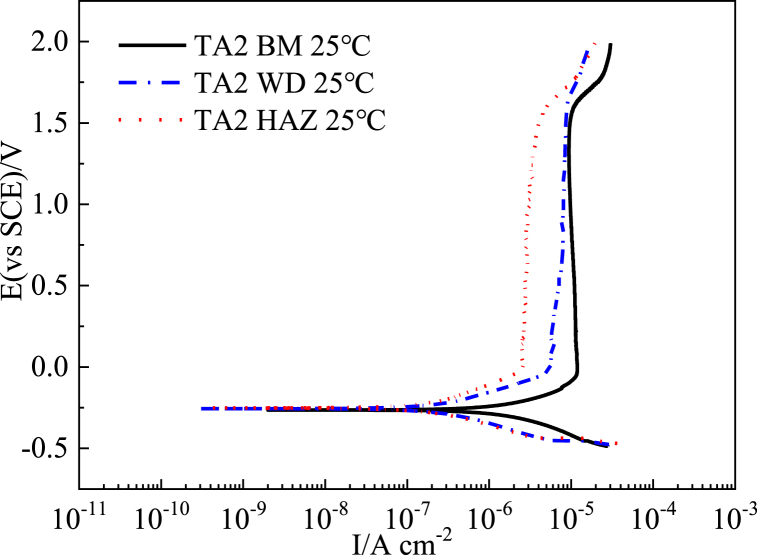
Polarization curves of TA2 base metal and TA2 weld and heat-affected zone metal in 3 wt% H_2_SO_4_ solution.

As seen from Fig. 3, the passivation current density (I _*pass*_) values of the TA2 base metal (10.3 μ A/cm^2^) in the 3.0 wt% H_2_SO_4_ flue gas simulation solution is higher than the corresponding weld metal (8.0 μ A/cm^2^) and heat-affected zone metal (1.8 μ A/cm^2^).And the corrosion current density (I _*corr*_), fitted by the extrapolation of Tafel curves, values of BM, WM and HAZ are 2, 0.35 and 0.16 μ A/cm^2^, respectively. The relationship between the three corrosion potentials is as follows: Heat-affected zone > weld zone > base metal. For passivated metal materials, the lower the passivation current density, the easier it is to passivate and protect the metal from further corrosion [18]. And the lower corrosion current density and higher corrosion voltage indicate that the metal has a low corrosion tendency. Therefore, it can be inferred that the corrosion resistance of TA2 base metal in the 3.0 wt%H_2_SO_4_ flue gas simulation solution is worse than the corresponding weld metal and heat-affected zone metal, resulting in a relatively higher corrosion rate. Zhou [6] also reached a similar conclusion in their experiment.

**Fig. 4:**
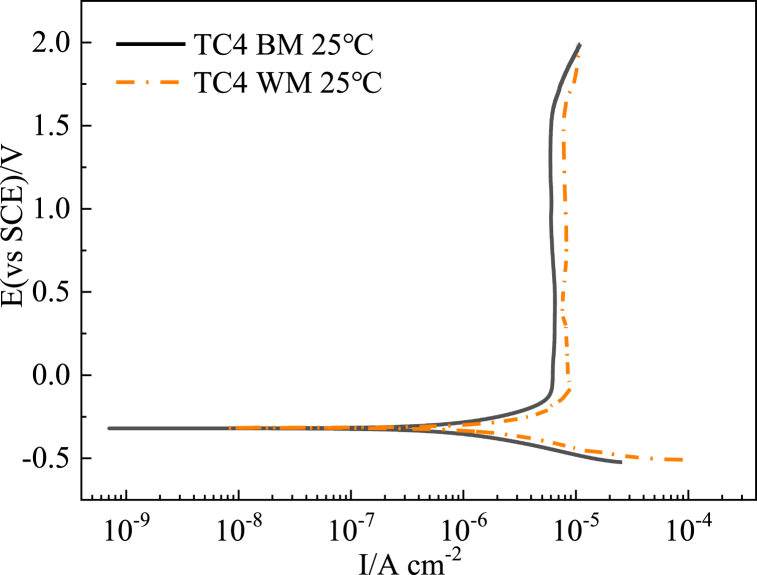
Polarization curves of TC4 base metal and TC4 weld metal in 3 wt% H_2_SO_4_ solution.

**Fig. 5:**
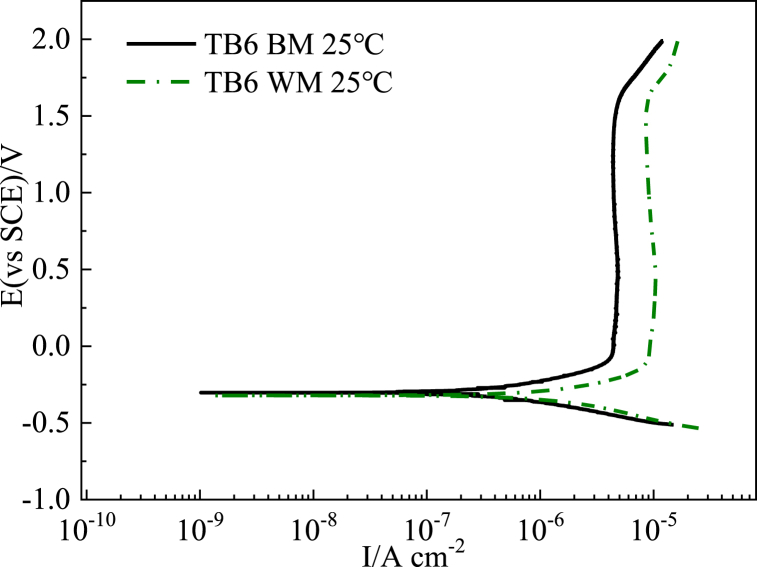
Polarization curves of TB6 base metal and TB6 weld metal in 3 wt% H_2_SO_4_ solution.

Comparing the corrosion behavior of the TC4 and TB6 titanium alloy base metals and weld metals, it can be seen from Fig. 4, Fig. 5 that the passivation current density of TC4 (6.0 μ A/cm^2^) and TB6 (4.3 μ A/cm^2^) the base metals in the 3.0 wt% H_2_SO_4_ flue gas simulation solution is lower than the corresponding weld metals (8.5 μ A/cm^2^ for TC4 WM and 8.0 μ A/cm^2^ for TB6 WM). Additionally, the corrosion potentials of the base metals are higher than that of the weld metals and both with a lower corrosion current density. Therefore, it can be inferred that the corrosion resistance of the TC4 and TB6 base metals in the 3.0 wt%H_2_SO_4_ flue gas simulation solution is better than of their corresponding weld metals, resulting in a relatively lower corrosion rate.

A sulfuric acid solution with a concentration of 3 wt% was selected to simulate the corrosion environment of chimney flue gas at 25 °C. Electrochemical impedance spectroscopy (EIS) was carried out on three kinds of titanium alloy base metal and corresponding weld metals, and TA2 heat-affected zone metals. The test results were carried out analyzed as below.

**Fig. 6:**
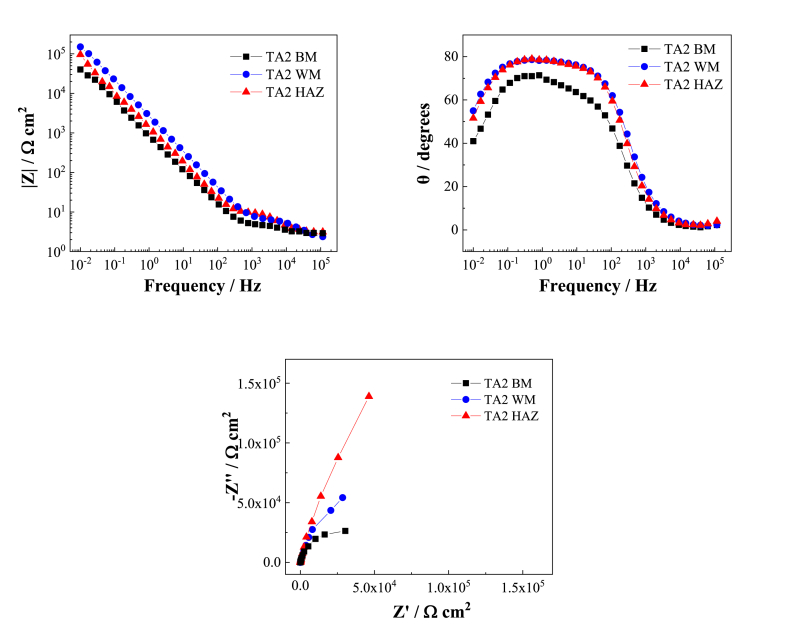
EIS spectra of TA2 base metal and TA2 weld and heat-affected zone metal in 3 wt% H_2_SO_4_ solution.

From the Bode diagram in Fig. 6, the low frequency impedance of TA2 base metal in the 3 wt% H_2_SO_4_ flue gas simulation solution is lower than that of the weld metal and heat-affected zone metal. Additionally, the slope of the low-frequency impedance is lower than that of the corresponding weld metal and heat-affected zone metal. From the phase angle diagram, it is found that the maximum phase angle of the weld metal is the same as the heat-affected zone, and both are larger than that of the base metal. This indicates a less stable passivation film with reduced resistance to ion penetration from the solution [19,20].

In the Nyquist diagram of Fig. 6, there is only one capacitor ring for the three regional metals. However, the capacitive reactance arc diameter of the base metal is smaller than that of the weld metal and the heat-affected zone metal. Therefore, it can be inferred that the surface passivation film of the TA2 weld metal and heat-affected zone metal in the 3.0 wt% H_2_SO_4_ flue gas simulation solution at 25 °C is more stable than that of the base metal, and their corrosion resistance is better than that of the base metal.

**Fig. 7:**
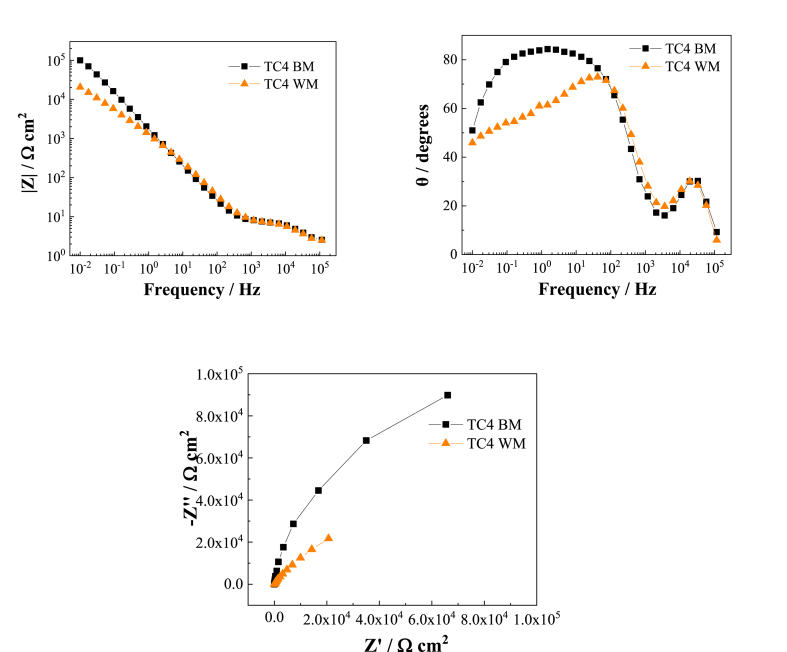
EIS spectra of TC4 base metal and TC4 weld metal in 3 wt% H_2_SO_4_ solution.

**Fig. 8:**
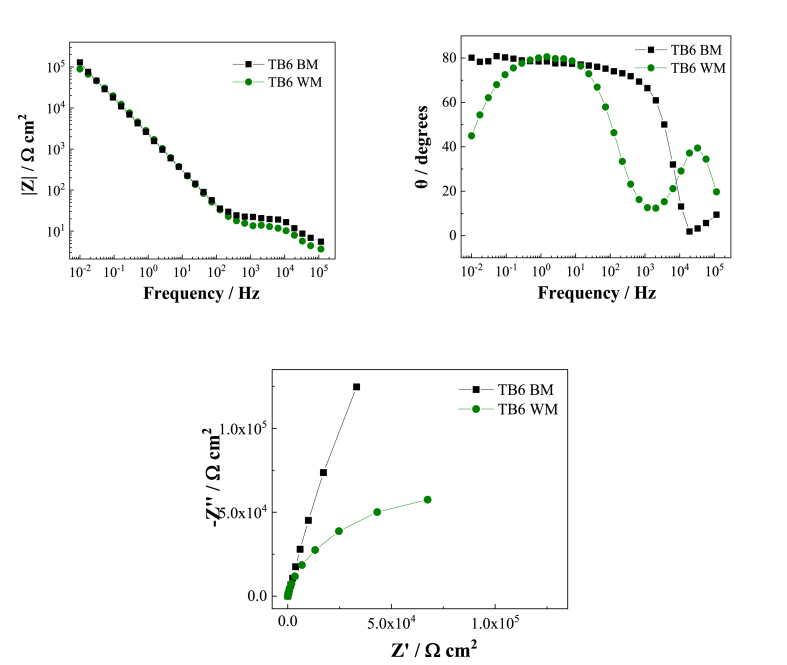
EIS spectra of TB6 base metal and TB6 weld metal in 3 wt% H_2_SO_4_ solution.

From Fig. 7, Fig. 8 Bode diagram, it can be seen that the low frequency impedance of TC4 and TB6 base metal in the 3.0 wt% H_2_SO_4_ flue gas simulation solution is higher than that of the corresponding weld metal, and the slope of the low frequency impedance is lower than Corresponding weld metal; it is found from the phase angle diagram that the maximum phase angle of the TC4 base metal is close to 90°, which is greater than the maximum phase angle of the corresponding weld metal at about 67°, so the passivation film of the TC4 base metal is more stable. Although the maximum phase angle of TB6 base metal is approximately the same as the maximum phase angle of weld metal, the phase angle at low frequency of the TB6 weld metal decreases significantly with decreasing frequency, while the TB6 base metal maintains a phase angle close to 90° and shows obvious capacitive behavior. This indicates that the TB6 base metal passivation is also more stable.

In the Nyquist diagrams in Fig. 7, Fig. 8, it can be seen that both the titanium alloy base material and the weld have only one capacitor ring, and the capacitive reactance arc diameter of the base material is significantly larger than the corresponding weld metal. , Which shows that the surface passivation film of the base metal of TC4 and TB6 two titanium alloys in the 3.0 wt% H_2_SO_4_ flue gas simulation solution is more stable than the corresponding weld metal, and the corrosion resistance of the two titanium alloy base metals The properties are better than the corresponding weld metal. The electrochemical impedance test results are consistent with the analysis conclusions of the potentiodynamic polarization test.

**Fig. 9:**
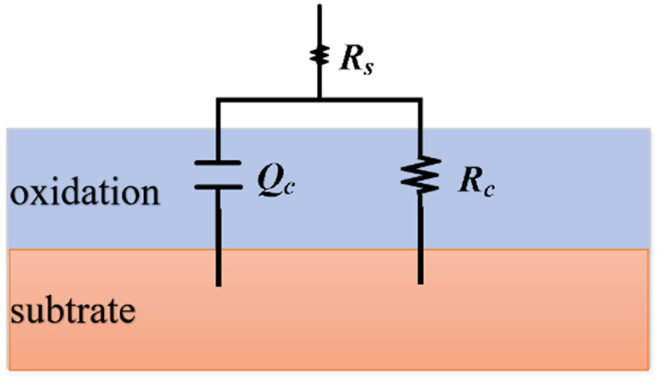
Equivalent circuit diagram of TC4 base metal and TC4 weld metal in 3 wt% H_2_SO_4_ solution.

**Fig. 10:**
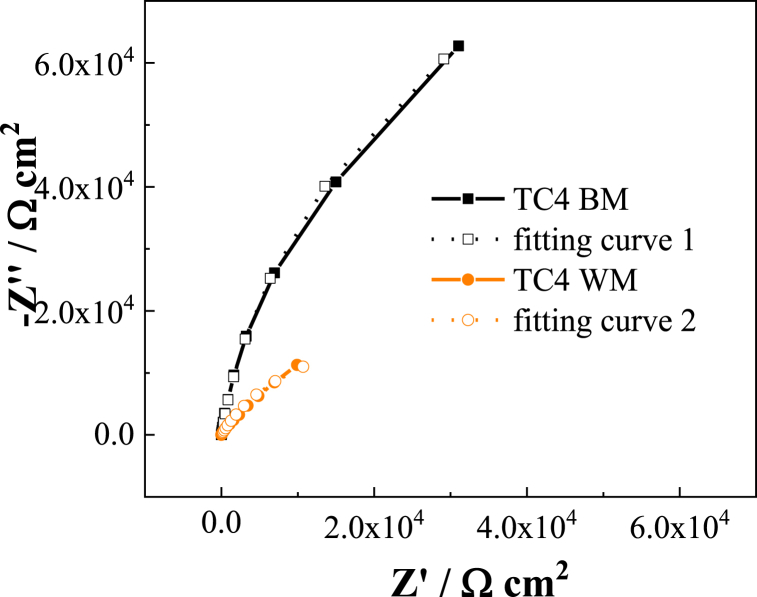
Equivalent circuit fitting EIS spectra.

The equivalent circuit model shown in Fig. 9 is used to fit the impedance data of the three types of titanium alloy base metal, corresponding weld metal, and TA2 heat-affected zone metals. As depicted, the fitting circuit represents the passivation film as an effective barrier layer. In the figure, *R*_*S*_, *R*_*C*_ and *Q*_*C*_ represent solution resistance, oxide film resistance and oxide film capacitance, respectively. The Nyquist plot fitting curve for the TC4 base metal and weld metal as shown in Fig. 10.

The fitting values of the equivalent circuit components are shown in Table 4. The passivation film in the TA2 weld zone and heat-affected zone metal shows higher impedance and lower capacitance than the base metal. It indicates that the metal in the TA2 weld area can inhibit the surface corrosion of titanium alloy more effectively than the base metal. In contrast, the corrosion resistance of TC4 and TB6 titanium alloys is opposite to that of TA2. The base metals of the two alloys exhibit significantly higher film resistance and lower film capacitance when fitted with circuit components. Thus, the base metals of TC4 and TB6 are more resistant to corrosion than their weld metals. This is consistent with the EIS test results.

**Table 4:** EIS equivalent circuit fitting results.

sample	R_S_/Ω cm^2^	R_C_/M cm^2^	CPE/10^−5^ Ω^−1^ S^n^ cm^2^	n
TA2 BM	1.02	0.0186	580	0.91
TA2 WM	12.54	0.0637	199	0.89
TA2 HAZ	29.12	0.0981	164	0.91
TC4 BM	6.14	0.1152	10	0.92
TC4 WM	3.98	0.0446	25	0.93
TB6 BM	16.85	0.1238	9	0.84
TB6 WM	11.05	0.0512	28	0.89

### 3.2 Characterization

Comparing the electrochemical test results, it is concluded that argon arc welding improves the corrosion resistance of TA2 welds but reduces the corrosion resistance of TC4 and TB6 titanium alloy welds. This significant difference requires further in-depth analysis. The following analysis results are based on a series of characterization methods (metallographic microscopy, XRD, SEM/EDS):

At the center of the argon arc welding seam, the grains in the TA2 welding seam area grew slowly, and almost half of the entire welding surface consist of fine dendritic grains. Additionally, the heat-affected zone near the weld has equiaxed grains that are uniformly distributed. This uniform structure prevents an increase in grain size in the heat-affected zone and weld zone, as grain boundaries are high-energy states, and larger grain boundaries introduce more dislocations and defects [21]. Refining the grain size can provide more active sites for the nucleation and growth of the passivation film [22], and a homogeneous structure helps reduce corrosion tendency [23]. Moreover, refining the dimensions of individual microcell cathodes can also significantly reduce electrochemical corrosion effects [24]. Therefore, the increase in grain size of TC4 and TB6 caused by argon arc welding promotes a decrease in corrosion resistance, while the TA2 weld increases corrosion resistance due to grain refinement.

In-depth analysis was conducted on the corrosion behavior of various titanium alloys and welds in a 3 wt% sulfuric acid flue gas simulation solution, considering factors such as microstructure, phase composition, and element content.

**Fig. 11:**
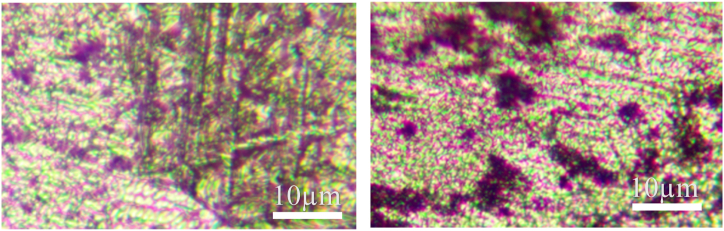
Microstructure morphology of TA2 base metal (left) and TA2 weld metal (right).

**Fig. 12:**
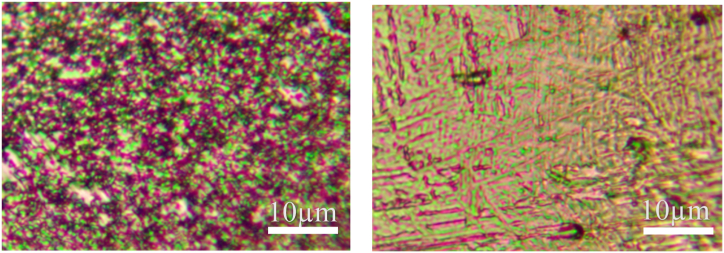
Microstructure morphology of TC4 base metal (left) and TC4 weld metal (right).

**Fig. 13:**
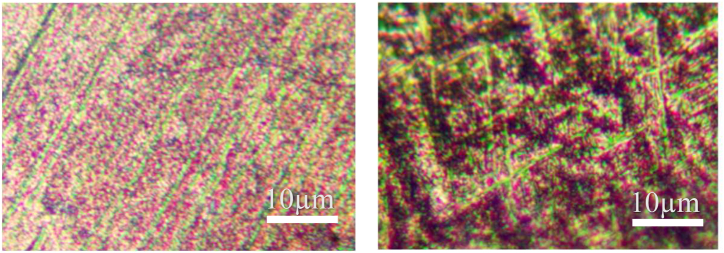
Microstructure morphology of TB6 base metal (left) and TB6 weld metal (right).

The microstructure of the base metal of the three titanium alloy materials and their corresponding weld metals under a metallographic microscope are shown in Fig. 11, Fig. 12, Fig. 13. Titanium alloys exhibit an allotropic crystal structure. At temperatures below the β phase transformation point, they possess a hexagonal close-packed crystal structure, while at temperatures above this point, they adopt a body-centered hexagonal crystal structure.

Fig. 11 illustrates the microstructure of the base metals of three titanium alloy materials and their corresponding weld metals under a metallographic microscope. After argon arc welding, some pores appear in the weld area of TA2, but small spherical equiaxed crystals are still observable. Fig. 12 reveals that TC4 displays densely distributed small spherical equiaxed primary α-phase grains and residual β-phase before welding. After welding, β-phase grains nucleate and grow during slow cooling or α′-phase formation due to martensite decomposition. The α′-martensite exhibits a needle-like morphology, typical of structures with an aspect ratio of about 10:1 [25]. The martensite transformation in the weld zone follows the grain growth mode, resulting in significant grain growth in TC4 weld metal. Similarly, Fig. 13 depicts significant grain growth in the weld area of TB6 after welding, forming columnar grains after complete martensite transformation. The structure morphology transitions from originally small α-phase crystal grains to larger columnar crystal grains, interlaced with each other.

From the analysis, it is evident that compared to the weld structure of TA2, the grains of the weld structure of TC4 and TB6 have undergone phase transformation. Compared with the fine equiaxed crystal of the base material, the grain size is obviously increased, forming a needle-like shape. Martensite grains or columnar grains, while the grains of TA2 will not be completely recrystallized during the welding process [26], the grain size has little change compared with the two titanium alloys TC4 and TB6 before and after welding.

**Fig. 14:**
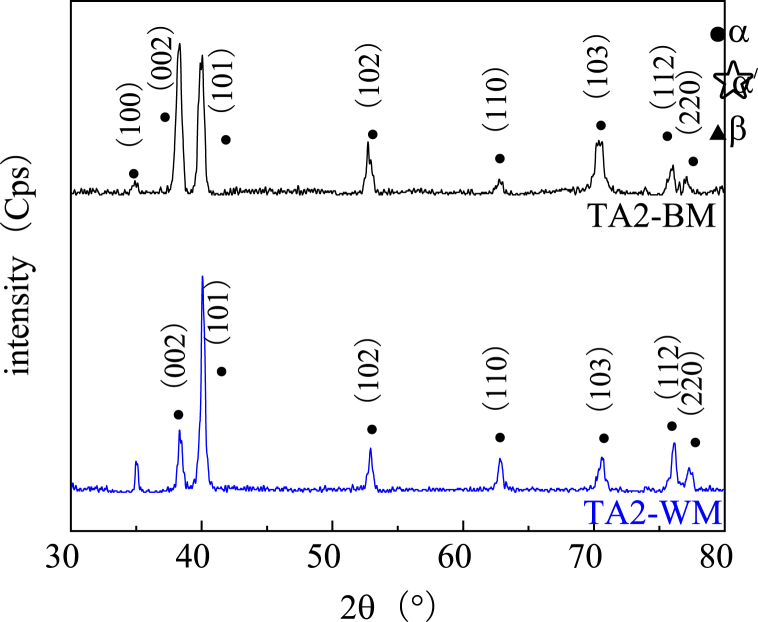
X-ray diffraction pattern of the TA2 joints in different regions.

**Fig. 15:**
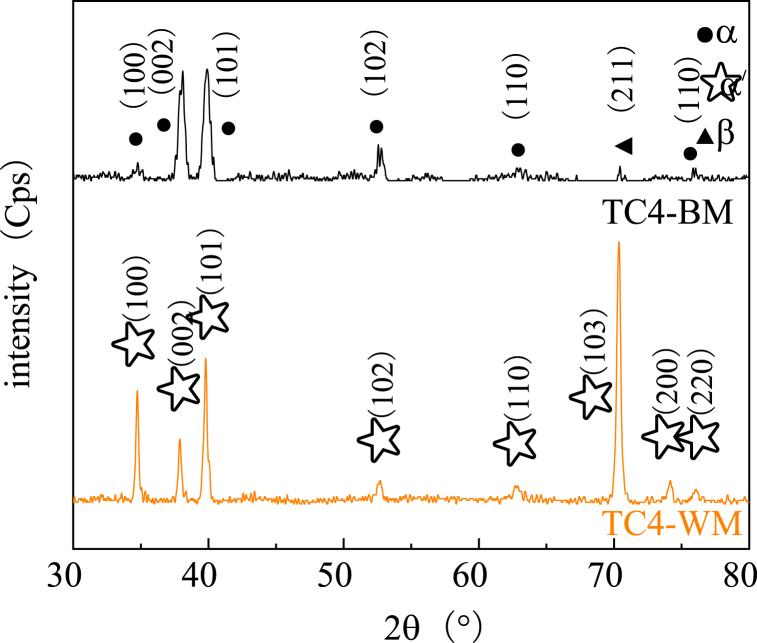
X-ray diffraction pattern of the TC4 joints in different regions.

**Fig. 16:**
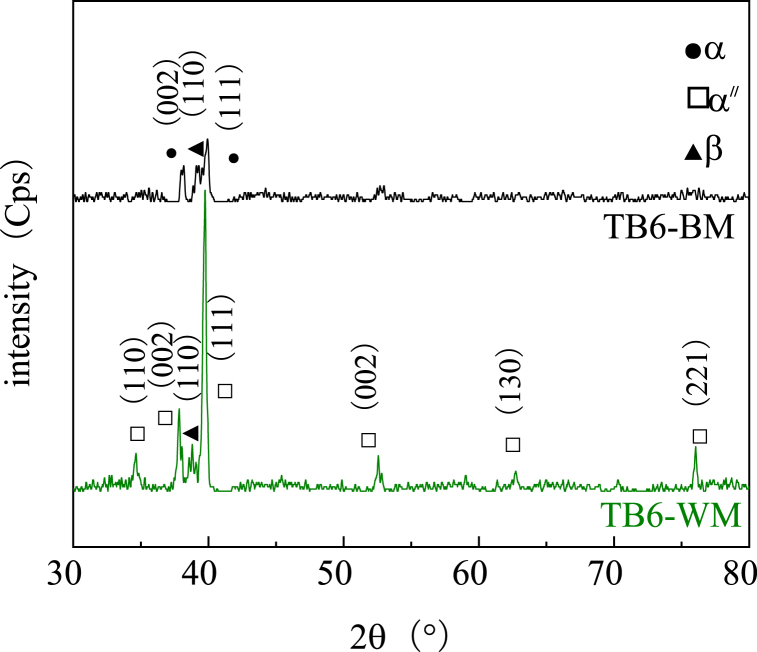
X-ray diffraction pattern of the TB6 joints in different regions.

In Fig. 14, Fig. 15, Fig. 16, the X-ray diffraction (XRD) patterns of the base metal and weld area of titanium alloys TA2, TC4, and TB6, respectively, are depicted. For TA2, compared to the base metal region, the intensity of (002) α-Ti is significantly reduced in the weld area, while (103) α-Ti shows a slight decrease. The peak strength of other phases experiences a slight increase. Additionally, the half-value width of the phases in the weld area remains similar to that of the base material. This suggests that the TA2 weld area retains fine equiaxed crystals, indicating that the welding process has not substantially altered the phase composition of TA2. Consequently, the grain size has not significantly increased, and both the weld metal and the base metal exhibit equiaxed and fine-grained structures.

Fig. 15 illustrates that the α phase is the dominant peak strength in the base metal of TC4, whereas the corresponding weld area is primarily composed of α′ phase. Compared to the base metal area, the XRD pattern of the TC4 weld area reveals a decrease in the peak intensity of (002) α-Ti phase, accompanied by a significant increase in the (100)α′-Ti and (103)α′-Ti peak. This observation further confirms that martensite transformation has occurred during the argon arc welding process of TC4 metal. Additionally, the narrowing of diffraction peak width in the weld area indicates an increase in grain size after welding. Consequently, the metal in the weld area undergoes a transformation from equiaxed fine grain to an acicular martensite structure, along with some columnar crystals [27].

In Fig. 16, the peak intensities of the metal phase composition in the TB6 weld area are compared with those of the base metal. It is observed that the peak intensities of (110) α''-Ti, (002) α''-Ti, (111) α''-Ti, and (221) α''-Ti have significantly increased in the TB6 weld area, while the peak intensities of other phase components show slight increases. Additionally, the half-width of the diffraction peak is markedly reduced. This, combined with the comparison of microstructure morphology in Fig. 13, indicates a significant increase in grain size after welding. The weld structure of TB6 exhibits notably larger columnar crystals, which are interlaced with each other. The TB6 weld metal surface has undergone complete martensite transformation during the welding process, resulting in the formation of columnar coarse crystals.

**Fig. 17:**
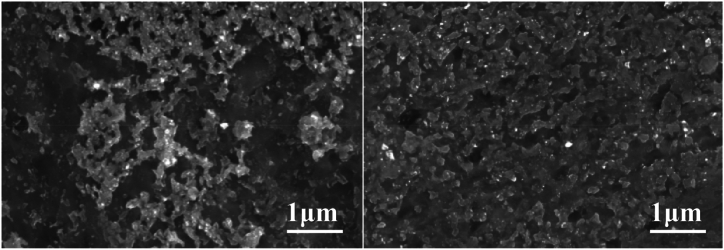
SEM comparison of TA2 base metal (left) and weld metal (right).

The scanning electron micrographs in Fig. 17 reveal that the equiaxed fine crystals of TA2 base metal did not exhibit significant growth before and after welding. Interestingly, the titanium alloy grains in the welding area became finer after welding, and their distribution became more uniform.

**Fig. 18:**
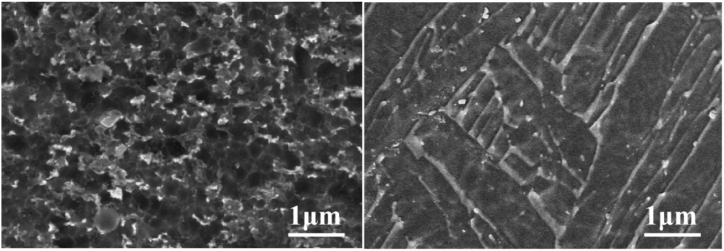
SEM comparison of TA2 base metal (left) and weld metal (right).

Fig. 18 illustrates noticeable changes in the crystal grains of TC4 base metal and weld metal. Before welding, the equiaxed fine crystals of the base metal area are apparent. However, after welding, the crystal grains undergo significant growth and adopt a needle-like structure.

**Fig. 19:**
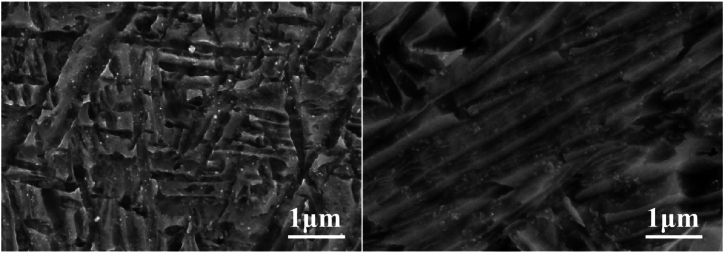
SEM comparison of TB6 base metal (left) and weld metal (right).

In the comparison of the scanning electron micrograph of the TB6 base metal and weld areas in Fig. 19, it is evident that the TB6 base metal grains display prominent grain boundaries before welding, and the grains are relatively fine. After welding, the grains in the weld area grow into columnar crystals, indicating complete martensite transformation.

The element content analysis of the three types of titanium alloy base metals and weld metals reveals interesting findings. According to Table 5, the weight percentage of oxygen atoms in TA2 weld metal (11.33 %) is notably higher than that of the base metal (6.44 %). This increase in oxygen content can be attributed to the role of oxygen as a stabilizing element of the α phase. Oxygen not only expands the range of α phase in the high-temperature zone but also contributes to solid solution strengthening [28]. Therefore, the grain size does not change significantly, and both the base metal and the weld metal exhibit equiaxed and fine-grained structures.

**Table 5:** The weight percentage and atomic percentage of TA2 base metal (left) and weld metal (right).

Elements	Weight	Atom	Elements	Weight	Atom
	(%)	(%)		(%)	(%)
C	2.36	7.85	C	2.21	6.82
O	6.44	16.09	O	11.33	26.26
Ti	91.20	76.06	Ti	86.46	66.92
Total	100.00		Total	100.00	

Additionally, the increase in oxygen percentage suggests that a certain oxidation reaction of titanium has occurred during the welding process, resulting in the production of titanium oxide. The generated titanium oxide is primarily rutile TiO_2_. Rutile titanium dioxide possesses an octahedral crystal structure and exhibits resistance to high and low temperatures, corrosion, and high strength [29]. Consequently, it may improve the corrosion resistance of the metal in the weld area.

From Table 6, it is evident that the weight percentage of oxygen atom in the TC4 weld metal (8.06 %) has increased compared to the base metal (5.72 %). However, the increase in oxygen content observed before and after TA2 welding, from 6.44 % to 11.33 %, is larger. This discrepancy suggests a potential β phase transition. When the oxygen content of the weld metal is too high, it can inhibit the β phase transition. Therefore, the smaller increase in oxygen content in TC4 weld metal compared to TA2 weld metal may indicate the occurrence of a β phase transition [30].

**Table 6:** The weight percentage and atomic percentage of TC4 base metal (left) and weld metal (right).

Elements	Weight	Atom	Elements	Weight	Atom
	(%)	(%)		(%)	(%)
C	3.03	9.69	C	3.23	9.91
O	5.72	13.73	O	8.06	18.60
Ti	82.65	66.28	Ti	79.63	61.35
Al	5.69	8.10	Al	5.55	7.58
V	2.92	2.20	V	3.53	2.56
Total	100.00		Total	100.00	

Referring to the SEM image of the TC4 weld in Fig. 18, the formation of acicular martensite after welding is observed, accompanied by significant changes in grain size. This observation supports the inference that the β phase transition does indeed exist in the TC4 weld area. This transition likely contributes to the observed changes in microstructure and grain size.

According to Table 7, the weight percentage of oxygen atoms in the TB6 weld metal (7.44 %) has increased compared to the base metal (3.35 %). Although the oxygen content is lower than that of the TA2 weld metal (11.33 %), the significant increase indicates a potential β phase transition. This inference aligns with the analysis of the SEM image of the TB6 weld in Fig. 19. In this image, grains transform into columnar structures after welding, exhibiting significantly larger grain sizes. Consequently, the base metal of TB6, characterized by equiaxed fine grains, contrasts with the weld metal, which consists of columnar coarse grains with complete martensite transformation. Additionally, the presence of a small amount of Fe in the base metal of TB6, acting as a β-phase stabilizing element, may influence the observed phase transitions.

**Table 7:** The weight percentage and atomic percentage of TB6 base metal (left) and weld metal (right).

Elements	Weight	Atom	Elements	Weight	Atom
	(%)	(%)		(%)	(%)
C	2.19	7.63	C	1.89	6.08
O	3.35	8.77	O	7.44	17.99
Ti	81.12	70.85	Ti	80.07	64.62
Al	2.62	4.06	Al	4.85	6.95
V	9.18	7.54	V	5.75	4.36
Fe	1.54	1.15			
Total	100.00		Total	100.00	

## 4 Conclusion

In a 3 % sulfuric acid simulated flue gas environment, the corrosion behavior of three titanium alloys—TC4, TB6, and TA2 were examined. Results indicate that the base metals of TC4 and TB6 exhibit lower passivation current densities and higher corrosion potentials than their corresponding weld metals. In contrast, the base metal of TA2 showed poorer corrosion resistance compared to its weld metal. This apparent difference in corrosion contrast behavior may be due to welding-induced changes in microstructural properties.

This paper conducts an in-depth analysis of various characterization methods for this apparently opposite corrosion behavior as follows:

Microscopic analyses (SEM/EDS, XRD, metallographic microscopy) suggest that the poor corrosion resistance of TB6 and TC4 welds may be attributed to β phase transformation. During argon arc welding, TC4 forms acicular martensite, which partially transforms into coarse columnar crystals, and TB6 undergoes a complete transformation into columnar crystals. This transformation results in poorer corrosion resistance of the weld metal compared to the base metal. In contrast, the grains of TA2 did not undergo significant recrystallization during the welding process, retaining their equiaxed fine grain structure.

These insights provide a reference for unifying the conclusions on the differences in corrosion behavior between titanium alloy base materials and welds and offer important theoretical and practical guidance for using these titanium alloys in challenging industrial environments, especially in the design and maintenance of titanium alloy chimneys.

## Data availability statement

Data will be made available on request.

## CRediT authorship contribution statement

**Caigan Che:** Writing – original draft. **Xuhui Zhao:** Resources. **Yu Zuo:** Writing – review & editing. **Yuming Tang:** Writing – review & editing.

## Declaration of competing interest

The authors declare that they have no known competing financial interests or personal relationships that could have appeared to influence the work reported in this paper:The authors declare the following financial interests/personal relationships which may be considered as potential competing interests: Caigan CHE reports equipment, drugs, or supplies and writing assistance were provided by 10.13039/501100007302Beijing University of Chemical Technology, China. Caigan CHE reports a relationship with Beijing University of Chemical Technology that includes:. If there are other authors, they declare that they have no known competing financial interests or personal relationships that could have appeared to influence the work reported in this paper.
